# Identification of Functional Mutations in GATA4 in Patients with Congenital Heart Disease

**DOI:** 10.1371/journal.pone.0062138

**Published:** 2013-04-23

**Authors:** Erli Wang, Shuna Sun, Bin Qiao, Wenyuan Duan, Guoying Huang, Yu An, Shuhua Xu, Yufang Zheng, Zhixi Su, Xun Gu, Li Jin, Hongyan Wang

**Affiliations:** 1 Chinese Academy of Sciences Key Laboratory of Computational Biology, Chinese Academy of Sciences and Max Planck Society (CAS-MPG) Partner Institute for Computational Biology, Shanghai Institutes for Biological Sciences, Chinese Academy of Sciences, Shanghai, China; 2 The State Key Laboratory of Genetic Engineering and MOE Key Laboratory of Contemporary Anthropology, School of Life Sciences, Fudan University, Shanghai, China; 3 Children’s Hospital of Fudan University, Shanghai, China; 4 Institute of Cardiovascular Disease General Hospital of Jinan Military Region, Jinan, China; 5 The Institutes of Biomedical Sciences, Fudan University, Shanghai, China; Oslo University Hospital, Norway

## Abstract

Congenital heart disease (CHD) is one of the most prevalent developmental anomalies and the leading cause of noninfectious morbidity and mortality in newborns. Despite its prevalence and clinical significance, the etiology of CHD remains largely unknown. GATA4 is a highly conserved transcription factor that regulates a variety of physiological processes and has been extensively studied, particularly on its role in heart development. With the combination of TBX5 and MEF2C, GATA4 can reprogram postnatal fibroblasts into functional cardiomyocytes directly. In the past decade, a variety of GATA4 mutations were identified and these findings originally came from familial CHD pedigree studies. Given that familial and sporadic CHD cases allegedly share a basic genetic basis, we explore the GATA4 mutations in different types of CHD. In this study, via direct sequencing of the *GATA4* coding region and exon-intron boundaries in 384 sporadic Chinese CHD patients, we identified 12 heterozygous non-synonymous mutations, among which 8 mutations were only found in CHD patients when compared with 957 controls. Six of these non-synonymous mutations have not been previously reported. Subsequent functional analyses revealed that the transcriptional activity, subcellular localization and DNA binding affinity of some mutant GATA4 proteins were significantly altered. Our results expand the spectrum of GATA4 mutations linked to cardiac defects. Together with the newly reported mutations, approximately 110 non-synonymous mutations have currently been identified in GATA4. Our future analysis will explore why the evolutionarily conserved GATA4 appears to be hypermutable.

## Introduction

Congenital heart disease (CHD) is a widespread birth defect that occurs in approximately 1∼5% of newborns and accounts for approximately one-tenth of all infant deaths [1]–[4]. During the past decade, remarkable progress has been made in elucidating the etiology of CHD, but the precise mechanisms involved in the majority of patients remain unknown.

In vertebrates, cardiogenesis is a very complex process that requires the accurate spatial and temporal cooperation of various regulatory factors. Disordered transcription factor interactions, altered hemodynamics, signal defects and microRNA dysfunction could all result in CHD [1]. The GATA4 zinc finger protein belongs to an evolutionarily conserved GATA family that consists of six members. It recognizes the nucleotide consensus sequence WGATAR in the promoter region of its downstream target genes [5]. Through interaction with specific cofactors, GATA4 regulates different physiological processes and integrates many signaling pathways [6]–[9]. GATA4 plays an especially critical role during several stages of cardiogenesis, from formation of the primitive heart tube to maturation of the four-chambered heart [10]. A recent study showed that in the presence of MEF2C and TBX5, GATA4 can induce cardiomyocyte differentiation and directly reprogram endogenous cardiac fibroblasts into cardiomyocytes by activating cardiac gene expression [11].

The accumulating evidences have shown that genetic defects in GATA4 play a vital role in the pathogenesis of CHD. Since the year it was identified as a genetic cause of septal defects [12], *GATA4* has been extensively screened for CHD-specific variations. All function-proved variations would help us in understanding the mechanisms underlying both the familial and sporadic non-syndrome cardiac defects. In this study, we conducted a population-based mutation screening of *GATA4* in 384 Chinese sporadic non-syndrome CHD patients and 760 matched controls. A total of twelve heterozygous non-synonymous mutations were found. Eight of these mutations were found exclusively in CHD patients, and six of them have not been previously reported. The functional analyses demonstrated that the GATA4 mutants A66T, A353T, E360G and a previously reported mutant T280M significantly changed the transcriptional activity of *ANF* luciferase reporter, and the C-terminal mutants A353T, E360G, G375R, S377G and A442V were partially located at the cytoplasm besides the nucleus. Gel shift assay showed that the DNA binding affinity of mutants A74D, G150W (*de novo*) and T280M reduced significantly compared to wild type GATA4.

According to published reports and the dbSNP database, there have been approximately 110 GATA4 non-synonymous mutations identified, including those identified in our study. Approximately 80% of these mutations are found in patients with septal defects. The functionally impaired GATA4 mutants identified in this study should contribute to the progress being made in the early diagnosis and prevention of congenital heart defects.

## Materials and Methods

### Ethics Statement

Protocols used in this study were reviewed and approved by the Ethics Committee of the School of Life Sciences, Fudan University. Written consent was obtained from the parents or guardians of the children patients prior to the study’s commencement.

### Study Subjects

Samples of the 384 CHD patients (185 males and 199 females) and 760 (362 males and 398 females) controls used in this study were consecutively recruited between August 2008 and February 2011 from the Cardiovascular Disease Institute of Jinan Military Command (Jinan, Shandong Province, China) and Children’s Hospital of Fudan University (Shanghai, China). CHD patients who were diagnosed with other syndromes or had a positive family history of CHD in a first-degree relative (parents, siblings and children) were excluded. If available, samples from the unaffected parents of the CHD patients were also collected. The controls were non-CHD outpatients from the same geographic area and during the same time period. The controls were also matched to the affected individuals in age and sex. The ethnicity of all the subjects is Han Chinese and they are genetically unrelated to each other.

We classified each of the 384 CHD cases into four broad categories as previously described [13]. Specifically, 34 (8.9%) had conotruncal defects, 275 (71.6%) had septation defects, 14 (3.6%) had right ventricular outflow tract obstruction, and 61 (15.9%) had other CHD defects (Table 1).

**Table 1:** Phenotypes of study subjects with congenital heart defects.

Classification	Number	Frequency (%)
CHD classification I		
Conotruncal defects	34	8.9
Septation defects	275	71.6
RVOTO	14	3.6
Other CHDs	61	15.9
CHD classification II		
Isolated CHD	324	84.4
Non-isolated CHD	60	15.6
Isolated CHD phenotype		
ASD	48	12.5
VSD	162	42.1
PDA	27	7.0
TOF	23	6.0

RVOTO, right ventricular outflow tract obstruction; ASD, atrial septal defect; VSD, ventricular septal defect; PDA,paten ductus arteriosus; TOF, tetralogy of Fallot.

Approximately 3–5 ml peripheral blood was collected from each test subject. Genomic DNA of each test subject was isolated from peripheral blood using conventional reagents.

### Sequencing and Genotyping of Human *GATA4*

The human *GATA4* gene (NM_002052.3), mapped to 8p23.1, consists of 6 exons that encode a 442 amino acid protein. For each of the collected 384 CHD cases, all exons and the intron-exon flanking regions of *GATA4* were amplified by polymerase chain reaction (PCR) for a mutation screen by sequencing. PCR primers were designed by the online software Primer3 and are listed in Table S1 (the reaction conditions are available upon request). PCR products were individually pretreated with a mixture of 1 unit ExoI and 1 unit SAP (TAKARA). Direct dye terminator sequencing of the purified PCR products was conducted using the ABI Prism BigDye system following the manufacturer’s instructions (ABI, Foster City, CA). Precipitation was done by 75% alcohol after second purification by 1 unit SAP. HiDi formamide was used in the subsequent denaturation, and finally the samples were subjected to sequencing using an ABI 3730XL sequencer. The results were analyzed by the Sequence Scanner and DNASTAR software.

The GATA4 mutations identified were confirmed by reverse sequencing of the independent PCR amplifications using the mutation carrier case DNA samples. The available DNA samples of the parents of CHD children carrying the identified mutations were also amplified and sequenced.

The 1000 Genomes Project data (http://www.1000genomes.org), which contains 197 Han Chinese people, was used as a phase one control to exclude benign GATA4 mutations. The first round CHD-specific GATA4 mutations identified with no record in the 1000 Genomes Project database were then genotyped in the 760 control samples using the SNaPshot method (ABI, Foster City, CA), and the results were analyzed by the Peak Scanner software. The CHD case specific mutations of GATA4 were finally identified after the comparison with the mixed controls (197+760).

### Plasmid Construction and Site Directed Mutagenesis

The construction of rat ANF-Luc has been previously reported [5], [14]–[16]. By pairwise sequence alignment, we confirmed the locations of the GATA4 binding sites in the human *ANF* (*NPPA*, NM 006172.3) promoter region. A 679 bp fragment of the human *ANF* promoter from −644 to +35 was PCR-amplified using pfu ultra (Stratagene, La Jolla, CA). The PCR products were cloned into the *Mlu*I and *BgI*II restriction sites of the pGL3-Basic vector (Promega) to produce ANF-Luc.

The full-length human *GATA4* cDNA was constructed and cloned into the Myc-tagged pCMV mammalian expression vector. Mutated complementary primers were designed, and PCR was performed using the full-length pCMV-Myc-GATA4 wild type plasmid as a template and the KOD polymerase enzyme (TOYOBO). After treating with *Dpn*I, the products were used to transform *E.coli* DH5α competent cells to select mutant GATA4.

To assess the impact of mutations in the C-terminal region on the structural and functional properties of GATA4, wild-type pEGFP-C1-GATA4 and mutated types were generated using the same method.

### Cell Culture and Luciferase Assay

Hela cells were cultured in Dulbecco’s minimum essential medium (DMEM,Gibco) supplemented with 10% fetal bovine serum (FBS,Gibco) and seeded in 24-well plates (1×10^5^) before transfection. Eighteen hours after plating, the cells were cotransfected with 200 ng of pGL3-ANF luciferase reporter, 200 ng of pCMV-Myc-GATA4 wild-type or mutant-type plasmids and 1 ng of pRL-TK (Promega) internal control plasmid using Lipofectamine™ 2000 (Invitrogen, Carlsbad, CA). Forty-eight hours after transfection, cells were collected and luciferase reporter levels were measured using the Dual-Luciferase Reporter Assay System according to the manufacturer’s instructions (Promega). The relative reporter activity was determined by normalizing the firefly activity to the *Renilla* activity. Protein expression levels were confirmed by western blot analysis, and each experiment was performed in triplicate for three times. The student’s *t*-test was used for statistical comparisons, and differences were considered to be significant if the *P*-value <0.05.

### Immunohistochemistry

Human cardiac myocytes cells (HCM, ScienCell) were cultured in cardiac myocyte medium (CMM). Forty-eight hours after the transient transfection of pEGFP-C1-GATA4 wild-type or mutant plasmids using Lipofectamine™ 2000, HCM cells were fixed with 4% formaldehyde/PBS, permeabilized with 0.2% Triton X-100/PBS, and stained with DAPI. Fixed cells were washed with 1× PBS 3 times for 5 minutes at the end of each step. A Laser Scanning Confocal Microscope was used to observe the results.

### Electrophoretic Mobility Shift Assay (EMSA)

We extracted nuclear proteins using Thermo Scientific NE-PER Nuclear and Cytoplasmic Extraction Reagents. The endogenous GATA4 expression of 293T, COS7, Hela and H1299 cells was detected by western blot using human GATA4-Specific Polyclonal antibody (Proteintech, Chicago, IL 60612, USA). Expression of GATA4 in Hela cells transfected with pCMV-Myc-GATA4 wild type and mutants were also detected using western blot.

Annealed oligonucleotides (5′-GCAGTTAACTGATAATGACACTGTG-3′) of human *dHAND* which correspond to the GATA element upstream of mouse *dHAND* used in previous studies were labelled with biotin [12], [17], [18]. EMSA was performed using LightShift Chemiluminescent EMSA kit (Pierce, Rockford, 1L 61101, USA). Binding reactions contained ddH_2_O, 2 ul of 10×binding buffer, 1µg of poly (dI-dC) and 2 µl of nuclear extracts. The reaction was incubated for 10 min at room temperature before 2ul (1 pmol) of biotin-labelled oligonucleotides were added. For competition analysis, 100-fold unlabled specific competitor or non-specific competitor was included in the binding reaction. A total of 20 ul reaction mix was incubated at room temperature for 20 minutes. The samples were loaded on a 6% nondenaturing polyacrylamide gel in 0.5×TBE.

## Results

### Identification of Human *GATA4* Mutations

By direct sequencing of the *GATA4* coding region, we identified 12 heterozygous non-synonymous mutations in 14 individuals out of the 384 patients with diverse forms of CHD (Table 2 and Table 3). None of these mutations were found in the 1000 Genomes Project sequencing results in the Chinese Han from Beijing (CHB) and Chinese Han from South (CHS) populations. In addition, we also found 8 synonymous mutations and 5 intronic variations in the flanking regions (Table 4).

**Table 2:** GATA4 non-synonymous variations only identified in 384 CHD patients.

Location	Nucleotide change	Amino acid change	Number	Cardiac defects	Parents carriers	New mutations/reported
Exon 1	c.196G>A	p.A66T	1	VSD, PDA	Father	[37]
	c.221C>A	p.A74D	1	PS	NA	Novel
	c.448G>T	p.G150W	1	TOF	De novo	Novel
Exon 2	c.628G>A	p.D210N	1	unknown	NA	Novel
	c.749T>A	p.I250N	1	VSD	Mother	Novel
Exon 5	c.1057G>A	p.A353T	1	TOF	Father	Novel
	c.1079A>G	p.E360G	1	VSD	NA	Novel
Exon 6	c.1325C>T	p.A442V	1	VSD	Mother	[25]

(Detailed clinical information for D210N was unclear, and “NA” means samples of the relative parents were unavailable.).

**Table 3:** GATA4 non-synonymous variations found in both CHD and control population.

Location	Nucleotide change	Amino acid change	Heterogeneity rate		Cardiac defects
			Patients(n = 384)	Controls(n = 760+197)	
Exon 1	c.487C>T	p.P163S	0.005 (2/384)	0.017 (13/957)	VSD,PS
Exon 3	c.799G>A	p.V267M	0.005 (2/384)	0.005 (4/957)	VSD,PDA
Exon 5	c.1123G>A	p.G375R	0.003 (1/384)	0.003 (2/957)	VSD
	c.1129A>G	p.S377G	0.003 (1/384)	0.005 (4/957)	VSD

**Table 4:** Synonymous and intronic variations identified in *GATA4.*

Location	Nucleotide change	Amino acid change	Number	Cardiac defects
Exon 1	c.99G>T	p.A33A	20/384	ASD,VSD, PDA, etc
	c.132G>T	p.V44V	1/384	VSD
	c.531C>A	p.A177A	1/384	VSD,PH
Intron 1	c.617-64G>A		1/384	
	c.617-16C>T		1/384	
Exon 2	c.678G>A	p.P226P	1/384	VSD
	c.723C>T	p.C241C	1/384	VSD
	c.744C>T	p.N248N	1/384	ASD,VSD
Intron 3	c.910-58T>A		17/384	
Exon 4	c.975G>A	p.L325L	1/384	ASD
Intron 5	c.1147-107A>G		1/384	
	c.1147-76C>A		1/384	
Exon 6	c.1326G>A	p.A442A	1/384	ASD

Four of these 12 CHD mutations (out of the first round compared with the 1000 Genomes Project data) (P163S, V267M, G375R and S377G) were also found in control individuals (Table 3), and had been previously reported (Table S2). Among the 8 mutations exclusively found in CHD patients, 6 of them (A74D, G150W, D210N, I250N, A353T and E360G) have not been previously reported, while the other 2 mutations (A66T and A442V) were reported in either previous studies or the dbSNP database. GATA4 G150W was a *de novo* mutation that was absent in both parents (Table 2).

We summarized all the non-synonymous mutations of GATA4, and found that currently, approximately 110 non-synonymous mutations have been identified in CHD cases within the coding region spanning the 3rd residue Gln to the 442nd residue Ala (Figure S1). These findings suggest that GATA4 is a hypermutable protein in CHD patients. To determine whether GATA4 is selectively constrained in the Chinese population, we calculated the nucleotide diversity π and sequence-based neutrality tests Tajima’s D using the public sequencing data of CHB and CHS from the 1000 Genomes Project [19]. *GATA4* is an essential gene; therefore, we restricted our comparison to 1475 essential genes [20]. The statistical analysis did not provide strong evidence showing that GATA4 is under positive or purifying selection in the Han Chinese population (Table 5).

**Table 5:** The Pi value and Tajimas’ D of GATA4 compared with 1475 essential genes based on the Han Chinese population data from the 1000 Genomes.

Regions	Population	N(Pi)	Pi value	N(Tajimas’ D)	Tajimas’ D
Transcriptregion	CHB	792	0.0007241	390	−0.24
	CHS	792	0.0007330	391	−0.34
Codingregion	CHB	792	0.0008428	149	−0.75
	CHS	792	0.0008486	149	−0.53

(N means the number of essential genes that have larger Pi value or Tajimas’ D than GATA4).

**Figure 1:**
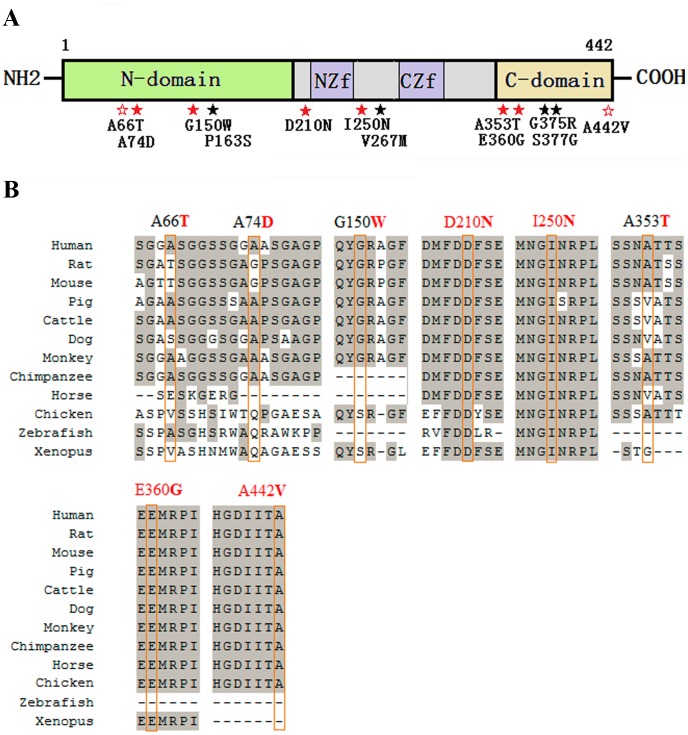
Distribution of the identified GATA4 mutations and multiple sequence alignment across species. (A) A schematic diagram of the GATA4 protein and the locations of the 12 non-synonymous mutations identified in this study (solid red star indicates a mutation that has not been previously reported; empty red star indicates a CHD-specific mutation that has been previously reported; solid black star indicates a mutation that is also found in controls). N-domain, N terminal domain; NZf, N terminal zinc finger domain; CZf, C terminal zinc finger domain; C-domain, C terminal domain. (B) Multiple sequence alignment of the GATA4 protein across different species. The result shows that residues 210 and 250 are highly evolutionarily conserved, and residues 360 and 442 are conserved in mammals.

As previously described, GATA4 contains two independent transcriptional activation domains in the N-terminal region, two highly conserved zinc finger domains, the interval basic domains and the C-terminal domain [3], [21]–[23]. The locations of the 12 mutations identified in this study are indicated in the GATA4 structural diagram in Figure 1A. Multiple sequence alignments indicate that among the 8 CHD specific mutations, only the residues 210, 250, 360 and 442 are highly conserved across species (Figure 1B). Even though these residues are highly conserved, it has been reported that the Glu located at residue 360 could be substituted by Gln or Gly, and that the Ala located at residue 442 could be substituted by Gly, Val or Thr (Table S2).

### Biochemical Analysis of GATA4 Mutations

**Figure 2:**
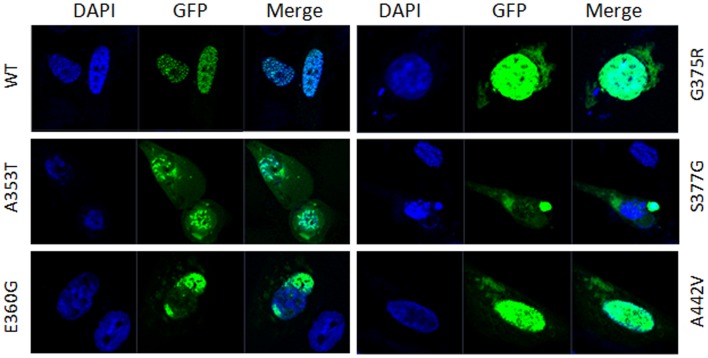
Subcellular location of GATA4 wild type and mutant proteins. GFP (green) represents the over expressed GATA4 protein, and DAPI (blue) represents the location of the nucleus. The subcellular localization of pEGFP-GATA4 wild-type (WT) and mutant-type proteins shows that wild-type GATA4 completely localized to the nucleus, while mutated GATA4 proteins were partially distributed in the cytoplasm besides the nucleus.

GATA4 is a nucleic transcription factor that has been reported to localize entirely in the nucleus [12], [18]. Five non-synonymous mutations A353T, E360G, G375R, S377G and A442V located in C-terminal region are adjacent to the nuclear localization signal (NLS) [21]. To determine whether these GATA4 mutants affect normal protein trafficking, which would then prevent GATA4 from properly functioning, immunofluorescence staining for GFP-tagged GATA4 was conducted in human cardiac myocytes cells. The results showed that wild-type pEGFP-GATA4 was exclusively located in the nucleus. The NLS adjacent mutants A353T, E360G, G375R, S377G and A442V, however, had partially abnormal localization patterns. A small amount of these GATA4 mutants appeared to be located in the cytoplasm (Figure 2). Similar results were also found in H1299 cells (data not shown). These results suggest that mutations adjacent to the NLS of hGATA4 impede normal nuclear localization.

**Figure 3:**
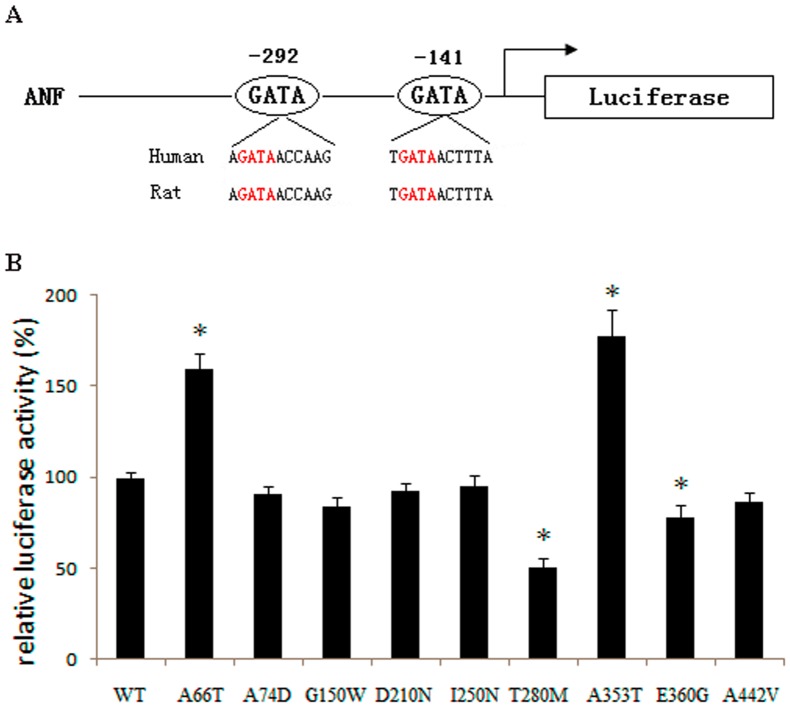
Relative luciferase activity of GATA4 wild type and mutant proteins in Hela cells. **(**A) Schematic of the human ANF-Luc. The region from the proximal −300 to −130 shows the conserved GATA transcription factor binding sites involved in regulating ANF expression. Two consensus GATA4 binding sites are highlighted in red. (B) Relative luciferase activity of wild type (WT) and CHD specific GATA4 mutant expression constructs that were co-transfected with ANF-Luc in Hela cells. Mean±SD are shown in the histogram. The relative luciferase activity for the A66T mutant was 160±8%, for the T280M mutant was 51±5%, for the A353T mutant was 178±14%, and for the E360G mutant was 78±6%. Student *t*-test was performed and four mutants caused significant transcriptional activation difference compared with wild-type GATA4 (*, *P*<0.05). Other mutants did not change the ANF-Luc activation significantly.

**Figure 4:**
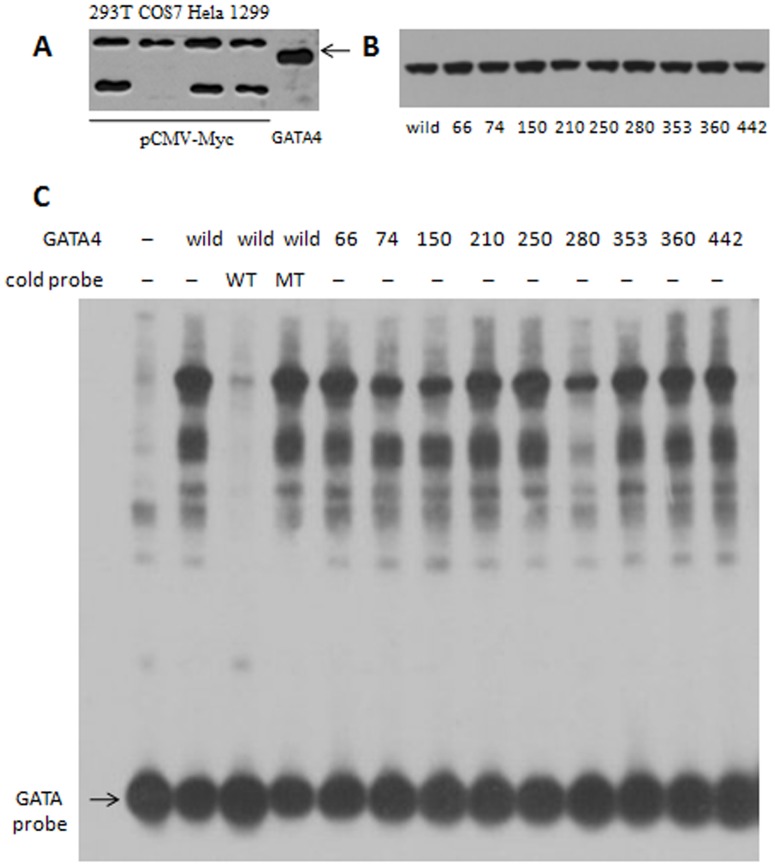
DNA binding affinity of GATA4 wild type and mutants. (A) Detection of the endogenous expression of GATA4 in 293T, COS7, Hela and H1299 cells transfected with pCMV-Myc empty vector. (B) Protein levels of nuclear extracts were kept equivalent between pCMV-Myc-GATA4 wild type and each mutant. (C) GATA4 A74D, G150W and T280M mutant proteins demonstrated abnormal DNA binding affinity compared to wild type GATA4 by using electromobility shift assay of biotin-labelled GATA binding element. 100-fold wild type cold oligonucleotides (WT) could compete for GATA4 binding while 100-fold cold mutant oligonucleotides (MT) failed to compete.

The human *ANF* gene promoter region contains two highly conserved GATA binding elements (Figure 3A) and is activated in a dose dependent manner by GATA4. The *in vitro* luciferase reporter assays showed that A66T and A353T significantly increased the ANF promoter activity; while T280M, a previously reported mutation identified in a Chinese CHD family [24], and E360G decreased the ANF-Luc reporter activity when compared with wild-type GATA4 (Student’s *t*-test: *P*<0.05). The other mutations tested did not significantly change the luciferase activity (Figure 3B). To further test the DNA binding affinity of GATA4 mutants, EMSA was performed with the GATA cis element upstream of human *dHAND.* Western blot result showed that GATA4 could not be detected in the nuclear extraction of the cells transfected with pCMV-Myc empty vector (Figure 4A). Expression of GATA4 in Hela cells transfected with pCMV-Myc-GATA4 wild type and mutants were kept equivalent (Figure 4B). The EMSA result showed that the DNA binding affinity of mutants A74D, G150W and T280M was clearly reduced compared to wild type GATA4 (Figure 4C).

## Discussion

GATA4 is essential for normal heart development, and a variety of mutations in this gene have been found in many cardiac defects. We found 12 heterozygous GATA4 mutations in a large panel of 384 sporadic CHD patients and 8 of them were found exclusively in CHD patients. Of these 8 mutations, 6 have not been previously reported. The prevalent frequency of GATA4 non-synonymous mutations specifically identified in sporadic CHD patients in our study is approximately 2.1% (8/384). These results are consistent with the 2.5% GATA4 mutation prevalence found in a previous study on 486 Chinese CHD patients [25]. The GATA4 mutation prevalence in Chinese CHD patients, however, is noticeably different from the 0.8% GATA4 mutation prevalence found in 628 American CHD patients [26]. Altogether, these results suggest that GATA4 non-synonymous mutations contribute to CHD differently between different ethnic populations (Table S2).

Even though GATA4 is a highly evolutionarily conserved protein that plays an essential role in normal heart development, the numerous mutations found indicate that GATA4 is a hypermutable protein in CHD patients. These findings appear to be in contrast to previously published data suggesting that GATA4 is a hypomutable protein [26]–[29]. Currently, approximately 110 non-synonymous mutations have been found in the GATA4 protein, including the mutations identified in this study. Approximately one third of the identified mutations are highly evolutionarily conserved, and 16 mutations are located in the well-conserved zinc finger domains (Figure S1). Some of the functionally important substitutions have even been detected several times in CHD cases (Table S2). For example, the mutation Cys292Arg located in the C terminal zinc finger domain of GATA4 was found in 31 DNA samples isolated from 68 formalin fixed malformed hearts with septal defects [23]. All of this evidence suggests that GATA4 mutations are frequently observed in CHD groups.

Our immunostaining results showed that wild-type GATA4 localized to the nucleus, while the GATA4 mutants A353T, E360G, G375R, S377G and A442V were partially detected in the cytoplasm, which suggests that these mutants do not properly localize to the nucleus. The alternative conformation or misfolding of these mutants may impede GATA4 protein transportation and trafficking. These mutations are not located in the previously identified NLS region [21], but they do affect the distribution of the protein. Similarly, mutations located outside of the identified NLS of TBX5 also impede nuclear localization [30], [31]. These results suggest that the nuclear localization of these transcription factors is affected by a wider region of the protein that is not limited to the traditional NLS region.

The luciferase assay revealed that the mutants A66T and A353T up-regulated transcriptional activity, but the increased activation ability could not be explained by DNA binding affinity (Figure 4C). The mutation A66T is located in the TAD1 domain (amino acids 1–77) of GATA4 [21], but it is not evolutionarily conserved (Figure 1B, Figure S2). Mutants A74D and G150W, which located in the TAD1 and TAD2 (amino acids 130–177) domains separately [21], did not change the transcriptional activity significantly but the DNA binding affinity were obviously changed (Figure 4C). The loss-of-function effect observed in the T280M mutant may be because this mutation affects the structural stability of the zinc finger and thus impairs the protein’s transcriptional activity and DNA binding affinity (Figure 3B and Figure 4C). The mutations A353T and E360G are located in the nascent C-terminal domain of the GATA4 protein, but they have an opposite effect on the regulation of ANF expression. The 353rd residue changes from a hydrophobic, nonpolar Ala to a hydrophilic polar Thr, while the 360th residue changes from an acidic, polar Glu to a neutral, nonpolar Gly. These changes in residue properties may be responsible for the different regulatory effects observed for these mutants. Furthermore, GATA4 always regulates ANF expression synergistically with other cofactors, such as GATA6, TBX5, NKX2-5, or SP1 [12], [32]–[35]. In this study, we ignored the influence of other factors and only focused on the effect of GATA4 mutations. Therefore, the *in vitro* results may not precisely reflect the *in vivo* regulation of ANF expression.

The relationship between the phenotypes of CHD and the genotypes of the GATA4 mutations is very complex, although it is interesting to note that GATA4 mutations are frequently associated with septal defects,such as ASD, VSD and AVSD (Table S2). Periodically, a mutation will be specifically associated with one type of CHD. For example, it has been reported that S52F and S358Del are only associated with ASD, one of the most common types of CHD [35], [36]. However, the same CHD phenotype may be caused by different GATA4 mutations, and the same GATA4 mutation may result in different phenotypes in different individuals, even individuals belonging to the same family [12]. We also observed that an affected child could inherit the same mutation from his or her unaffected parents. The different CHD cases, however, may be the result of several factors, such as epigenetic regulation, genetic and environmental factors, and even the personalized genetic signature or age difference. In our study, the questionnaire data collected from the parents of children with CHD revealed that the mothers of these children may have caught a cold, had a fever, lived in a newly decorated house, or worked in an unclean environment, such as an electronics factory. We cannot exclude the possibility that these types of factors may influence the probability of offspring inheriting CHD, but we are unable to make accurate correlations at this time because these data have no matched control.

Finally, we need to address the hypermutable and hypomutable characteristics found in the GATA4 mutations. The large number of GATA4 non-synonymous mutation count historically identified in CHD patients suggests that it looks like a hypermutable protein. Moreover, most of the genetic results from CHD studies are based on blood samples (Table S2). This strategy, however, may not detect all of the disease associated mutations [4], especially the severe lethal mutations. Therefore, these results may not reveal all of the genetic alterations. But the general ratio of the identified GATA4 mutations in CHD patients is less than 3% as discussed before, which suggests that it is a hypomutable protein. So we further compared the mutation ratio of GATA4 with other essential genes using the Han Chinese population data from the 1000 Genomes Project and the results did not provide strong evidence that GATA4 is under positive or purifying selection in the Han Chinese population. Thus the hypermutable or hypomutable feature is a relative term based on different circumstances. The hypomutable feature is defined based on the mutation ratio, while the hypermutable feature comes from the large number of GATA4 mutations identified in CHD patients.

Why GATA4 mutations are enriched in CHD patients obviously is an interesting question. We first needed to determine whether the frequency of GATA4 mutations is higher than the frequency of mutations in other essential genes, which may help in explaining the phenomenon of enrichment. The results indicate that there is no difference in the mutation frequency for GATA4 compared with other essential genes (Table 5). This finding does not support the possibility that the hypermutable GATA4 is the result of a higher mutation frequency. If there were correlations between the more severe CHD symptoms and the more conserved or functionally important GATA4 mutations, then it would be reasonable for us to conclude that the conserved GATA4 gene mutations will result in a severe phenotype, such as CHD, and that many CHD patients will have several GATA4 mutations. It is difficult to make these correlations, however, because the classifications/manifestations of CHD are complex and only limited sample sizes have been reported.

The transcriptional regulatory pathways involved in cardiac morphogenesis are extremely complex, and the effects exerted by the critical genetic transcriptional factors participating in these networks are still being elucidated. From a clinical perspective, the identification of novel and functional GATA4 mutations could be potentially useful as risk predictors in the molecular diagnosis of CHD.

## Supporting Information

Figure S1 Geogrphical distribution of GATA4 mutations and conservation analysis.
(PDF)

Figure S2 Multiple sequence alignment of GATA 4, 5, 6 across species.
(PDF)

Table S1 GATA4 primers used in PCR amplification.
(DOCX)

Table S2 Detailed information of the identified GATA4 mutations so far.
(XLSX)
