# Supplementary material for: Identification of Functional Mutations in GATA4 in Patients with Congenital Heart Disease
Source: PLoS One. 2013 Apr 23;8(4):e62138. doi: 10.1371/journal.pone.0062138 (PMC3633926; doi:10.1371/journal.pone.0062138)
Supplement: Figure S1 — Geogrphical distribution of GATA4 mutations and conservation analysis. (PDF) [file pone.0062138.s001.pdf]

http://consurf.tau.ac.il/results/1333293377/Multicolored_Seq.php

ConSurf Results

```
1          11         21         31         41
MYQSLAMAAN HGPPPGAYEA GGPGAFMHGA GAASSPVYVP TPRVPSSVLG
eeeebebbee eeeeeeeeee eeeebbbbbb eeeeeebebb eeebeebbeb
ff                f                   ff  s    f

51         61         71         81         91
LSYLQGGGAG SASGGASGGS SGGAASGAGP GTQQGSPGWS QAGADGAAYT
beebeeeeee eeeebeeeee ebebbeeeee eeeeeeeebb eeeeeeeeee
  f                                         f

101        111        121        131        141
PPPVSPRFSF PGTTGSLAAA AAAAAAREAA AYSSGGGAAG AGLAGREQYG
eeeeeeebeb eeeeeeeeee eeeeebeebb ebebbbbbbb bebebeeeee
      f           f         f                    f

151        161        171        181        191
RAGFAGSYSS PYPAYMADVG ASWAAAAAAS AGPFDSPVLH SLPGRANPAA
eeeeebeeeb eeeeebbebb ebbbbbbbbe eeebeebebe bbeeeeeeee
 f      f   f  f        s                 f

201        211        221        231        241
RHPNLDMFDD FSEGRECVNC GAMSTPLWRR DGTGHYLCNA CGLYHKMNGI
eeeebebbee eeebeebbeb eeeebebbee eeebebbbbb bebebeeeee
f              ffssf  ff fsf sff fffsf s ss sfsf ffff

251        261        271        281        291
NRPLIKPQRR LSASRRVGLS CANCQTTTTT LWRRNAEGEP VCNACGLYMK
eeeebeeeee eeebeeebbbb beebeeeeee bbeeeeeeee bbebbbbbbe
ffff  ff f  f sff s   sffs f fff sssfff ffff ssfsssss f

301        311        321        331        341
LHGVPRPLAM RKEGIQTRKR KPKNLNKSKT PAAPSGSESL PPASGASSNS
beeeebebbe eeeebeeeee eeeeeeeeee eeeeeeeeee eeeeeeeeee
sf ffsfssf ffffsfffff fff   f

351        361        371        381        391
SNATTSSSEE MRPIKTEPGL SSHYGHSSSV SQTFSVSAMS GHGPSIHPVL
eebeeeeeee eeeeeeeeee eeeeeeeeee bebbbbeebe eeeeeeeeee
                f  f               f

401        411        421        431        441
SALKLSPQGY ASPVSQSPQT SSKQDSWNSL VLADSHGDII TA
bebeeeeeee eeeeeeeeee ebeeebbbbb bbbeeeeebb ee
   f ff                 f          s         ff
```

Variants marked above the sequence (in order of appearance):

Q3P, A6V, G16C, Q19E, G21V, H28D, H28Y, A32P, V39L, R43W, S46Del, S52F, G64E, A66T, A66P, G69D, S70T, A74D, P87S, S90R, G93A, D95E, P111fs, T114fs, 118-119insA, 125-126insAA, A131G, G150W, R151H, A152P, S157C, S160T, P163R, P163S, S191G, M207T, F208L, D210N, F211L, G214S, E216D, G221R, M223T, P226Del, R229S, G234S, N239D, N239S, Y244C, M247T, N248S, I250N, R252P, I255T, R260Q, L261P, R266X, V267M, N273S, T277I, T280M, R283H, N285K, A286V, E289K, C292R, A294V, G296C, G296S, G296R, H302R, M310V, R311W, Q316E, K329N, A346V, A353T, T354A, S358Del, E359Del, E359K, E360Q, E360G, M361V, R362C, R362H, G369V, H376Q, S377G, G375R, S377N, V380M, G391A, H392R, P394T, P394A, L403M, P407Q, A411V, T420N, D425N, N428T, S429T, L430V, V431G, L432S, H436Y, D438Y, A442G, A442T, A442V

The conservation scale:

1	2	3	4	5	6	7	8	9

Variable — Average — Conserved

e - An exposed residue according to the neural-network algorithm.
b - A buried residue according to the neural-network algorithm.
f - A predicted functional residue (highly conserved and exposed).
s - A predicted structural residue (highly conserved and buried).
X - Insufficient data - the calculation for this site was performed on less than 10% of the sequences.
