# Supplementary material for: Identification of Functional Mutations in GATA4 in Patients with Congenital Heart Disease
Source: PLoS One. 2013 Apr 23;8(4):e62138. doi: 10.1371/journal.pone.0062138 (PMC3633926; doi:10.1371/journal.pone.0062138)
Supplement: Figure S2 — Multiple sequence alignment of GATA 4, 5, 6 across species. (PDF) [file pone.0062138.s002.pdf]

CLUSTAL 2.1 MULTIPLE SEQUENCE ALIGNMENT

File: D:/New_LifeScience/collaborations/WangHongyan/GATA1 to GATA6/GATA345[illegible] Date: Fri Mar 23 22:31:26 2012


```
GATA-4_Human                   ------------------------------------------------------------------------------------------------------------------------------------------------------
ENSPTRG00000019993_Chimpanzee  ------------------------------------------------------------------------------------------------------------------------------------------------------
ENSBTAG00000005425_Cow         ------------------------------------------------------------------------------------------------------------------------------------------------------
ENSRNOG00000010708_Rat         ------------------------------------------------------------------------------------------------------------------------------------------------------
ENSMUSG00000021944_Mouse       ------------------------------------------------------------------------------------------------------------------------------------------------------
ENSGALG00000016662_Chicken     ------------------------------------------------------------------------------------------------------------------------------------------------------
ENSXETG00000000673_Frog        ------------------------------------------------------------------------------------------------------------------------------------------------------
ENSTRUG00000012112_J_Puffer    ------------------------------------------------------------------------------------------------------------------------------------------------------
ENSTNIG00000016369_T_Puffer    ------------------------------------------------------------------------------------------------------------------------------------------------------
ENSDARG00000035759_Zebrafish   ------------------------------------------------------------------------------------------------------------------------------------------------------
GATA-5_Human                   ------------------------------------------------------------------------------------------------------------------------------------------------------
ENSPTRG00000013711_Chimpanzee  ------------------------------------------------------------------------------------------------------------------------------------------------------
ENSCAFG00000012709_Dog         ------------------------------------------------------------------------------------------------------------------------------------------------------
ENSMUSG00000015627_Mouse       ------------------------------------------------------------------------------------------------------------------------------------------------------
ENSRNOG00000036732_Rat         ------------------------------------------------------------------------------------------------------------------------------------------------------
ENSGALG00000005352_Chicken     ------------------------------------------------------------------------------------------------------------------------------------------------------
ENSTNIG00000002769_T_Puffer    ------------------------------------------------------------------------------------------------------------------------------------------------------
ENSDARG00000017821_Zebrafish   ------------------------------------------------------------------------------------------------------------------------------------------------------
GATA-6_Human                   ----MALTDGGWCLPKRFGAAG-ADASD-SRAFPAREPSTPPSPISSSSSS---CSRGGERGPGGASNCGTPQLDTEAAAGPPARSLLLSSYASHPFGAPHGPSAPGVAGPGGNLSSWEDLLLFTDLDQAATASKLLWSSR---GAKLSP   138
ENSBTAG00000005734_Cow         ----MALTDGGWCLPKRLGAAPPADASD-SGAFPAREPSTPPSPISSSSSSC--CSRGGERGPGGASNCRTPQLDTEAAAGPTARSLLLSPYASHPFGAPHGPSAPGISGPAGALSSWEDLLLFADLDQAATASKLLWSSR---GAKLSP   140
ENSMUSG00000005836_Mouse       ----MALTDGGWCLPKRFGAAA-ADAGD-SGPFPAREPSSPLSPISSSSSS---CSRGGDRGPCGASNCRTPQLDAEAVAGPPGRSLLLSPYASHPFAAAHGAAAPGVAGPGSALSTWEDLLLFTDLDQAATASKLLWSSR---GAKLSP   138
ENSRNOG00000023433_Rat         ----MALTDGGWCLPKRFGAAA-ADAGD-SGPFPAREPSSPLSPISSSSSS---CSRGGDRGPCGASNCRTPQLDTEAVAGPPGRSLLLSPYTSHPFAAAHGAAAPGVAGPGSALSTWEDLLLFTDLDQAATASKLLWSSR---GAKLSP   138
ENSTRUG00000012674_J_Puffer    -----------LLTPTLYGKCSWMDLGENSWSMVKREVSSS--PGSPAEQT---YLPGDSRRDGPTSDDLRTP-PSDLDALGHRRPDGRSLHSYVHFGHHN-----------NTLTTVEDIPLFTDLDQGG---KLVLPGG----AHKTG   115
ENSTNIG00000014837_T_Puffer    ---------------------SWMDLGENSWSMVKREVSSS--PGSPAEQS---YLPGDSRRDGPASDDLRTP-PSDLDALGHRRPDGRSLHSYVHFGHHN-----------NTLSTAEDIPLFTDLDQGG---KLVLPGG----AHKAG   105
ENSDARG00000035831_Zebrafish   -----------------------MDLGDNSWSMVKREVSSSSSPGSPAEQS---YLNTDRR------DRLRSPAPTHLDPVGSRRAEGRPLHSYVHFGHPN-----------NALPSSDDVTLFTDLDQGN---KLIISGGHPRETHKGG   104
ENSXETG00000003144_Frog        MSWERHPQDSRLRTPISQRPHTWMDLNEDSWSGGTRLSPANSFFPCPIKQSPGTSPPPSPCSQLGSSECLRTEPDRGPHTIS---AEGRPAPSYHPLG----------------LSHPEDLILFRDLDHIN---KLIVPNR---ETRYSS   125
FBgn0003117_Fly                ------------------------------------------------------------------------------------------------------------------------------------------------------
                               1.......10........20........30........40........50........60........70........80........90.......100.......110.......120.......130.......140.......150
```

```
                                            :                    ..               .    ..           *                                     .
GATA-4_Human                   ---------MYQSLAMAANHGPPPGAYEAGGPGAFMHGAGA-------ASSPVYVPTPR-VPSSVLGLSY-LQGGGAGSASGGASGGSSGGAASGAGPGTQQGSPGWSQA-GADGAAYTP---------------------PPVSP----   106
ENSPTRG00000019993_Chimpanzee  ---------MYQSLAMAANHGPPPGAYEAGGPGAFMHGAGA-------ASSPVYVPTPR-VPFFVLGLSY-LQGGGAGSASGGASGGSSGGAASGAGPGTQQGSPGWSQA-GADGAAYTP---------------------PPVSP----   106
ENSBTAG00000005425_Cow         ---------MYQSLAMAANHGPPPGPYEAGGPGAFMHGAGA-------ASSPVYVPTPR-VPSSVLGLSY-LQGGGGAASGAASGGSSGAAPSGAGPGTQQGSPGWSQA-GAEGAAYTP---------------------PPVSP----   106
ENSRNOG00000010708_Rat         ---------MYQSLAMAANHGPPPGAYEAGGPGAFMHSAGA-------ASSPVYVPTPR-VPSSVLGLSY-LQGGGSGAASGATSGGSSGAGPSGAGPGTQQGSPGWSQA-GAEGAAYTP---------------------PPVSP----   106
ENSMUSG00000021944_Mouse       ---------MYQSLAMAANHGPPPGAYEAGGPGAFMHSAGA-------ASSPVYVPTPR-VPSSVLGLSY-LQGGGSAAAAGTTSGGSSGAGPSGAGPGTQQGSPGWSQA-GAEGAAYTP---------------------PPVSP----   106
ENSGALG00000016662_Chicken     ---------MYQSLAMAANPGPP--SYEGAG--GFMHGAAA-------AS-PVYVPTTR-VPSMLPSLPY-LPSSGSSQQASPVSSHS-----------------IWTQP-GAESAAYNPG---------------SS--HPPVSP----    88
ENSXETG00000000673_Frog        ---------MYQSIAMAANHGPP--AYEGAG--GYMHSATA-------ATSPVYVPTTR-VSSMIPSLPY-LQTSGSSQQGSPVSGHN-----------------MWAQA-GAEPSAYNPG---------------TS--HPPVSP----    89
ENSTRUG00000012112_J_Puffer    ---------MYQGI-MTSNHGPP--SYEAG----FLHNTSA-------ATSPVYVPTTRVVTPMIPALPY-LQTPGASQQSSPVSSHS-----------------AWAQP-GSDSVSSYS--------------------HSPVSP----    84
ENSTNIG00000016369_T_Puffer    ---------MYQGI-MSSSHGPP--SYEAG----FLHSTSA-------GTSPVYVPTTRVVTPMIPALPY-LQTPAASQQSSPASSHS-----------------AWTQP-GSEPVSSYS--------------------HSPVSP----    84
ENSDARG00000035759_Zebrafish   ---------MYQGVTMAANHGAA--SYESG----FLHNS----------TSPVYVTPTR-VTPMIQALPY-LQAP---QQSSPASGHS-----------------GWAQP-GVETTSYNSS---------------TGHHHSPVS-----    82
GATA-5_Human                   ---------MYQSLALAASPRQAAY-ADSGS---FLHAPG--------AGSPMFVPPAR-VP---SMLSY-LSGCEPSPQPPELA-----------------ARPGWAQTATADSSAFG-----------------PGSPHPPAAHPPGA    90
ENSPTRG00000013711_Chimpanzee  ---------MYQSLALAASPRQAAY-ADSGS---FLHAPG--------AGSPMFVPPAR-VP---SMLSY-LSGCEPSPQPPELA-----------------ARPGWAQTATADSSAFG-----------------PGSPHPPAAHPPGA    90
ENSCAFG00000012709_Dog         ---------MYPSLALAPSPGQAAY-TDSGA---FLPAPA--------AGSPVFVPPAR-VP---PMLPY-LPPCEPGPQAPGRG-----------------AHPGWAQ--AADSAPFG-----------------PGSPPPPAAPPPAA    88
ENSMUSG00000015627_Mouse       ---------MYQSLALAQSPGQGTY-ADSGA---FLHSSG--------TGSPVFVAPTR-MP---SMLPY-LPSCEPGSQAPALA-----------------AHSSWTQAVAADSSAFG-----------------SGSPHPPAAHPPGA    90
ENSRNOG00000036732_Rat         ---------MYQSLALAQSPGQGTY-ADSGP---FLHSSG--------AGSPVFVAPTR-VP---SMLPY-LPSCEPGSQPPALA-----------------AHSSWTQAVSAESSAFG-----------------SGSPHPPATHPPGA    90
ENSGALG00000005352_Chicken     ---------MYQGLALAPNHGQSAYSHDSGN---FLHSS---------AGSPVYVPTTR-VPSVLQTLPY-LQSCEP--HQSHLG-----------------NPPGWAQS-SGETTAFN-----------------AGSPHPPSG-----    85
ENSTNIG00000002769_T_Puffer    ---------MYQSLALSPN--QPPYPHDTGT---YLHPP---------ASSPVYVPSSR-VPAVLPTLPY-LQPCEAPPQSHGLGGHHGLGGHHG-----LGGHHGWPPA-SADGPPFP-----------------PSSPLPPHG-----    97
ENSDARG00000017821_Zebrafish   ---------MYSSLALSSN--PSPYAHDSGN---YIHPS---------ASSPVYVPTTR-VPAMLQTLPY-LQTCESSHQAHGIS-----------------SHHAWPQT-GTDNSSFN-----------------PGSPHPPPG-----    85
GATA-6_Human                   FAPEQP-EEMYQTLAALSSQGPAAY-DGAPGG--FVHSAAAAAAAAAAASSPVYVPTTR-VGSMLPGLPYHLQGSGSGPANHAGG---------------AGAHPGWPQA-SADSPPYGSGGGAAGGGAAGPGGAGSAAAHVSAR-----   262
ENSBTAG00000005734_Cow         FAPEQP-EEMYQTLAALSS-GPAAY-DGSPGG--FVHSAAAAAAAAAAASSPVYVPTTR-ANKMLPGLPY-LQGAGGGPANHAGG---------------AGAHPGWPQA-SADSX----------------------------------   233
ENSMUSG00000005836_Mouse       FAAEQP-EEMYQTLAALSSQGPAAY-DGAPGG--FVHSAAAAAAAAAAASSPVYVPTTR-VGSMLSGLPY-LQGAGSGPSNHAGG---------------AGAHPGWSQA-SADSPPYG-GGGAAGGGAAGPGGAGSATAHASAR-----   260
ENSRNOG00000023433_Rat         FAAEQP-EEMYQTLAALSSQGPAAY-DGAPGG--FVHSAAAAAAAAAASS-PVKN-TTR-VGSMLPGLPY-LQGAGSGPSNHAGG---------------AGAHPGWPQA-SADSPPYG-GGGAAGGGAAGPGGAGSATAHASAR-----   258
ENSTRUG00000012674_J_Puffer    LLVDP--TDMYQTLAIAAAQSQTGY-DSSSGG--YMHSNP---------NSPVYVPSSR-VGSMIPSLSY-LQAGGSAQPNHA-----------------VSSHSVWSQS-TTESPSYS-----------------TGSPHTSSR-----   209
ENSTNIG00000014837_T_Puffer    LLVDP--TDMYQTLAIAAAQSQSGY-DSSPGG--YMHSNP---------NSPVYVPSSR-VGSMIPSLSY-LQAGG-AQPNHA-----------------VSSHSVWSQS-TTESPSYS-----------------TGSPHASSR-----   198
ENSDARG00000035831_Zebrafish   LIVDP--TDMYQTLAIAAAQSQAGYNDTPSAG--YMHSNP---------TSPVYVPTSR-VGTMIPNLSY-LPPGVSTQPSHA-----------------VSSHSVWSQP-APESPSYS-----------------TGSPHTSNR-----   199
ENSXETG00000003144_Frog        ALPEPPAGDMYQTLTITAAQGPLGY-DHSQGT--FMHSAA---------SSPVYVPTSR-VGSMLTSISY-LQGTGASQGAHS-----------------VNNH--WSQA-TSEGSSFS-----------------SSSPHTSSR-----   219
FBgn0003117_Fly                -------------MGILLSDGDSTSDQQSTRDYPHFSGDYQN---VTLSAASASTSASASATHVAAVKMYHSSAVAAYTDLAAAG---------------------SAASAGVGVGVSG-------------------------------    82
                               .......160.......170.......180.......190.......200.......210.......220.......230.......240.......250.......260.......270.......280.......290.......300
```

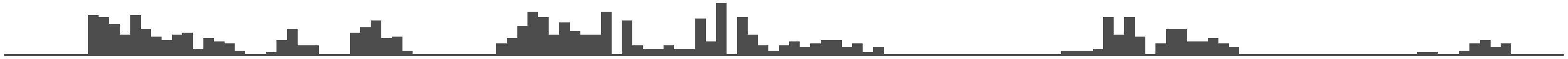

CLUSTAL 2.1 MULTIPLE SEQUENCE ALIGNMENT

File: D:/New_LifeScience/collaborations/WangHongyan/GATA1 to GATA6/GATA34[illegible]Date: Fri Mar 23 22:31:26 2012


```
                                                                                                                                              :  :                       . ::    :.*
GATA-4_Human                  -RFSFPGTTGSLAA--AAAAAAAREAAAYSSGGGAAGAGLAGREQYGR---------------------AGFAG----------SYSSPYPAYMADVGASWAAAAAASAGPFDSPVLHSLPGRA-NPAA-RHPN----LDMFDDFS-EGR  215
ENSPTRG00000019993_Chimpanzee -RFSFPGTTGSLAA--AAAAAAAREAAAY--------------------------------------------------------------------------AAAASAGPFDSPVLHFLPGRA-NPAA-RHPN----LDMFDDFS-EGR  172
ENSBTAG00000005425_Cow        -RFSFPGTTGSLAA--AAAAAAAREAAAYSSGSGAAGAGLAGREQYGR---------------------AGFAG----------SYSSPYPAYMADVGASWAAAAAASAGPFDSPVLHSLPGRA-NPAA-RHPN----LDMFDDFS-EGR  215
ENSRNOG00000010708_Rat        -RFSFPGTTGSLAA--AAAAAAAREAAAYSSSGGAAGAGLAGREQYGR---------------------PGFAG----------SYSSPYPAYMADVGASWAAAAAASAGPFDSPVLHSLPGRA-NPA--RHPNL---VDMFDDFS-EGR  215
ENSMUSG00000021944_Mouse      -RFSFPGTTGSLAA--AAAAAAAREAAAYGSGGGAAGAGLAGREQYGR---------------------PGFAG----------SYSSPYPAYMADVGASWAAAAAASAGPFDSPVLHSLPGRA-NPG--RHPNL---VDMFDDFS-EGR  215
ENSGALG00000016662_Chicken    -RFSFSTSTPIPST--SSRDAAAAYSSSLSLSAN-------GREQYSR----------------------GFGS----------SYSSPYPAYVSP-----EMATTWTSSPFDSPMLHNLQSRG-TPAAARHANI---AEFFDDYS-EGR  186
ENSXETG00000000673_Frog       -RFTFSSSPPISAA--SNR--EVPYSSPLAISAN-------GREQYGR----------------------ALGA----------TYSSPYPAYMSP-----DMGATWTASPFDSSMLHNLQNRA-GPAASRHPNI----EFFDDFS-EGR  184
ENSTRUG00000012112_J_Puffer   -RFAFSTSPPLSSGVAAARETAASYTNALNISAN-------GREHYGP---------------------RSLGG----------SYHSPYTPYMSP-----NVGGTWPASPFDSPVLHGLQSGT-ATGAARHPN----IDLFDDYA-EGR  184
ENSTNIG00000016369_T_Puffer   -RFSFSTSPPLS-------ETAASYTNPLNISAN-------GREHYGS---------------------RSLGG----------SYHSPYTPYMSP-----NVGGTWPASPFDSPVLHGLQSGT-PTGARGTQTSFILIDLFDDFA-EGR  181
ENSDARG00000035759_Zebrafish  -RFTFSTSPPLTSG-----RDTATYT---------------SREHFGS---------------------RALSG----------SYHSGYPAYVSP-----NIGASWTASHFDSSVLHSLQTGG-AAAAR-HPN----LEFFDDLG-EGR  168
GATA-5_Human                  TAFPFAHSPSGPGSGGSAGGRDGSAYQGALLP----------REQFAA------------------PLGRPVGT----------SYSATYPAYVSP-DVAQS----WTAGPFDGSVLH--------GLPGRRPTFV--SDFLEEFPGEGR  187
ENSPTRG00000013711_Chimpanzee TAFPFAHSPSEPGSGGSAGGRDGSAYQGALLP----------REQFAA------------------PLGRPVGT----------SYSATYPAYVSP-DVAQS----WTAGPFDGSVLH--------GLPGRRPTFV--SDFLEEFPGEGR  187
ENSCAFG00000012709_Dog        TAFPFPHSPSGPGGG--AGARDGGAYQGALLA----------REQYPA------------------PLGRPVGA----------SYPAAYPAYVSP-EGAPA----WTSGPFEGSVLHGLQSRP-AGLPGRRATFV--SDFLEELPGEGR  190
ENSMUSG00000015627_Mouse      TTFPFAHSPPGSGSGGSAGVRDGGAFQGALLA----------REQYPT------------------PLGRPMGA----------SYPTTYPAYMSS-DVAPS----WTSGAFDSSILHGLQARP-GGLPGRRTSFV--PDFLEEFPGEGR  194
ENSRNOG00000036732_Rat        TAFPFAHSPSGSGSGGSAGVRDGAAFQSALLA----------REQYPT------------------ALGRPMSA----------SYPTTYPAYVSP-DVAPS----WTSGPFDSSILHGLQGRP-GGLPGRRTTFV--PDFLEEFPGEGR  194
ENSGALG00000005352_Chicken    --FSYPHSPP----GSSPPGRDG-AYQGPLLLGGGG------REQYGN------------------ALVRSVNG----------SYSSPYPAYVTP-ELPPS----WTAGHFESSVLHSLQTRQ-AALPGRRSTF----EYLEEFPGDGR  184
ENSTNIG00000002769_T_Puffer   --FSYSHSPP----GSGPSGREA-GYQGALVLGNGAR-----ADAYGG------------------ALVRTFGG----------SYSSPY-AYMSP-EMATS---SWTPGPFESGVPK-NRKIR-PASLGSDRSL----NLMDDGSTEGR  196
ENSDARG00000017821_Zebrafish  --FSYSHSPP----VSSSTGRDA-AYQNPLMLSNGGR-----ADQYGS------------------ALVRSVGG----------SYSSPYAAYMSP-EMATS----WTPGPFDGGMIG-LQGRQ-GTLPGRRSSI----DMLDDLPCEGR  184
GATA-6_Human                  --FPYSPSPP----MANGAAREPGGYAAAGSGG---------AGGVSGGGSSLAAMGGREPQYSSLSAARPLNGTYHHHHHHHHHHPSPYSPYVGA-PLT---PA-WPAGPFETPVLHSLQSRAGAPLPVPRGPS---ADLLEDLS-ESR  388
ENSBTAG00000005734_Cow        ----------------------------------------------------------------------------------HHHHPSAYSPYVGA-PLT---PA-WPAGPFETPVLHSLQSRAGAPLPVPRGPG---TDLLEDLP-ESR  292
ENSMUSG00000005836_Mouse      --FPYSPSPP----MANGAARDPGGYVAAGGTG---------AGSVSGGGGSLAAMGGREHQYSSLSAARPLNG---TYHHHHHHHHPTYSPYMAA-PLT---PA-WPAGPFETPVLHSLQGRAGAPLPVPRGPS---TDLLEDLS-ESR  382
ENSRNOG00000023433_Rat        --FPYSPSPP----MANGAARDPGGYVAAGGAG---------AGSVSGGGGSLAAMGGREHQYSSLSAARPLNG---TYHHHHHHHHPTYSPYMGA-PLT---PA-WPAGPFETPVLHSLQSRAGAPLPVPRGPS---ADLLEDLS-ESR  380
ENSTRUG00000012674_J_Puffer   --FHYPPSPP----MNNGTPRDGGYTNTLNVGS---------RDQYG--------------------LSRPLSG----------TYASPYSPYVAP-QLSQLPSP-WGGGPFDNTMLHTLQSRG-APL-IRGPNGV--SDILDDMG-ESR  307
ENSTNIG00000014837_T_Puffer   --FHYPPSPP----MNNGTPRDGGYSNTLNVSS---------REQYG--------------------LSRPLSG----------TYASPYSPYVAP-QLSQLSSP-WGGGPFDNTMLHTLQSRG-APL-IRGPNGV--SDILDDMG-ESR  296
ENSDARG00000035831_Zebrafish  --FHYSPSPP----MNNGTSRDTSYTTPLNVNS---------RDQY---------------------LSRPISG----------SYPSPYPPYVTP-QLSQLPAA-WPAGPFENSMLHSLQSRS-APLAIRGPNG----DLLEEMV-ESR  295
ENSXETG00000003144_Frog       --YHYSPSPP----MANGSTRDTGYSSSLTVSG---------RDQYTP-------------------LARPLNG----------SYGSPYTPYMAP-QLT---SA-WPAGPFDNTMLHSLQSRG-APINVRGAPG----DVLDELP-ESR  314
FBgn0003117_Fly               -YHQQAVNAPVYVPSNRQYNHVAAHFGSAAAQN-----------------------------------AWTTEG-----------FGSAHAQFYSP----------------NAAVMMGSWRSAYDPSGFQRSSP--YESAMDFQFGEGR  167
                              .......310.......320.......330.......340.......350.......360.......370.......380.......390.......400.......410.......420.......430.......440.......450
```

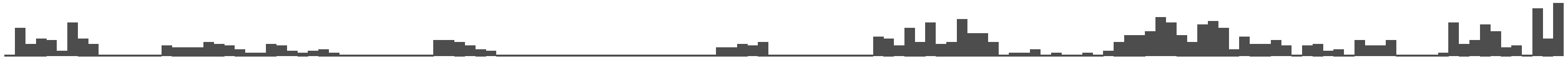

```
                              ******::.*****:*****:******** **** .***  *.:*    ..:** **.*:** * .*** * * :*********** ***** :**: :*:.*  *** *.   .
GATA-4_Human                  ECVNCGAMSTPLWRRDGTGHYLCNACGLYHKMNGINRPLIKPQRR---LSASRRVGLSCANCQTTTTTLWRRNAEGEPVCNACGLYMKLHGVPRPLAMRKEGIQTRKRKPKNLNKSKTPAAPSGSESLPPASGASS-NSSNATTSSS--E  359
ENSPTRG00000019993_Chimpanzee ECVNCGAMSTPLWRRDGTGHYLCNACGLYHKMNGINRPLIKPQRR---LSASRRVGLSCANCQTTTTTLWRRNAEGEPVCNACGLYMKLHGVPRPLAMRKEGIQTRKRKPKNLNKSKTPAAPSGSESLPPASGASS-NSSNATTSSS--E  316
ENSBTAG00000005425_Cow        ECVNCGAMSTPLWRRDGTGHYLCNACGLYHKMNGINRPLIKPQRR---LSASRRVGLSCANCQTTTTTLWRRNAEGEPVCNACGLYMKLHGVPRPLAMRKEGIQTRKRKPKNLNKSKTPAGPSGSESLPPATSASS-SSSSVATSSS--E  359
ENSRNOG00000010708_Rat        ECVNCGAMSTPLWRRDGTGHYLCNACGLYHKMNGINRPLIKPQRR---LSASRRVGLSCANCQTTTTTLWRRNAEGEPVCNACGLYMKLHGVPRPLAMRKEGIQTRKRKPKNLNKSKTPAGPPG-ESLPPSSGASS-NSSNATSSSSSE  360
ENSMUSG00000021944_Mouse      ECVNCGAMSTPLWRRDGTGHYLCNACGLYHKMNGINRPLIKPQRR---LSASRRVGLSCANCQTTTTTLWRRNAEGEPVCNACGLYMKLHGVPRPLAMRKEGIQTRKRKPKNLNKSKTPAGPAG-ETLPPSSGASSGNSSNATSSSSSE  361
ENSGALG00000016662_Chicken    ECVNCGAMSTPLWRRDGTGHYLCNACGLYHKMNGINRPLFKPQRR---LSASRRVGLSCANCHTTTTTLWRRNAEGEPVCNACGLYMKLHGVPRPLAMRKEGIQTRKRKPKNLNKTKTPAGPSSSESLTPTT-----SSTSSSSSATTTE  328
ENSXETG00000000673_Frog       ECVNCGAMSTPLWRRDGTGHYLCNACGLYHKMNGINRPLIKPQRR---LSASRRVGLSCANCHTTTTTLWRRNAEGEPVCNACGLYMKLHGVPRPLAMKKEGIQTRKRKPKNLSKSKTPTGQNSSDSLTPST-----SSTNSAG-----E  321
ENSTRUG00000012112_J_Puffer   ECVNCGAMSTPLWRRDGTGHYLCNACGLYHKMNGINRPLIKPQRR---LSASRRVGLSCTNCHTTTTTLWRRNAEGEPVCNACGLYMKLHGVPRPLAMKKEGIQTRKRKPKNLNKIQP--GTPGGEGTPVTP-------SSTPRTSAASE  322
ENSTNIG00000016369_T_Puffer   ECVNCGAMSTPLWRRDGTGHYLCNACGLYHKMNGINRPLIKPQRR---LSASRRVGLSCTNCHTTTTTLRR-NAEGEPVCNACGLYMKLHGVPRPLAMKKEGIQTRKRKPKNLNKTQP--GTPGAEGTPATP-------SSTPRTSAASE  318
ENSDARG00000035759_Zebrafish  ECVNCGAMSTPLWRRDGTGHYLCNACGLYHKMNGINRPLVKPQRR---LSASRRVGLSCTNCQTTTTTLWRRNAEGEPVCNACGLYMKLHGVPRPLAMKKEGIQTRKRKPKNISKTKP--GSSEGQSAISAV-------NSSP-----TE  301
GATA-5_Human                  ECVNCGALSTPLWRRDGTGHYLCNACGLYHKMNGVNRPLVRPQKR---LSSSRRAGLCCTNCHTTNTTLWRRNSEGEPVCNACGLYMKLHGVPRPLAMKKESIQTRKRKPKTIAKARGSSGSTRNASASPSAVA---STDSSAATSKAKP  331
ENSPTRG00000013711_Chimpanzee ECVNCGALSTPLWRRDGTGHYLCNACGLYHKMNGVNRPLVRPQKR---LSSSRRAGLCCTNCHTTNTTLWRRNSEGEPVCNACGLYMKLHGVPRPLAMKKESIQTRKRKPKTIAKARGSSGSTRNASASPSAVA---STDSSAATSKAKP  331
ENSCAFG00000012709_Dog        ECVNCGALSTPLWRRDGTGHYLCNACGLYHKMNGVNRPLVRPQKR---LSSSRRAGLCCTNCHTTTTTLWRRNADGEPVCNACGLYMKLHGVPRPLAMKKESIQTRKRKPKSVVKTKSNSGGLGNGTASPPSVP---DPESPAATLKPKP  334
ENSMUSG00000015627_Mouse      ECVNCGALSTPLWRRDGTGHYLCNACGLYHKMNGVNRPLVRPQKR---LSSSRRSGLCCSNCHTATTTLWRRNSEGEPVCNACGLYMKLHGVPRPLAMKKESIQTRKRKPKNPAKIKGSSGSTANTTASSPTLL---NSESSATTLKAES  338
ENSRNOG00000036732_Rat        ECVNCGALSTPLWRRDGTGHYLCNACGLYHKMNGVNRPLVRPQKR---LSSSRRSGLCCSNCHTATTTLWRRNAEGEPVCNACGLYMKLHGVPRPLAMKKESIQTRKRKPKNPAKIKGSSGSTANSTASSPTLL---NTESSATTLKAES  338
ENSGALG00000005352_Chicken    ECVNCGAMSTPLWRKDGTGHYLCNACGLYHKMNGINRPLK-PQKR---LSSSRRAGLCCTNCHTTNTTLWRRNAEGEPVCNACGLYMKLHGVPRPLAMKKESIQTRKRKPKNITKGKTSTGSTTSATNSPSSIT---NSDS-TVTLKSEP  326
ENSTNIG00000002769_T_Puffer   ECVNCGSVSTPLWRRDGTGHYLCNACGLYHKMNGSNRPLVRPQRR---PAPSRRAGLCCTNCGTSTTTLWRRNAEGEPVCNACGLYMKLHGVPRPLAMKKESIQTRKRKPK-WPKSRNAAGPGISDSSSPSSLA---PSGH-ASSIKSEP  338
ENSDARG00000017821_Zebrafish  ECVNCGSISTPLWRRDGTGHYLCNACGLYHKMNGINRPLIKPQKRL--QSTSRRAGLCCTNCHTSTTTLWRRNAEGEPVCNACGLYMKLHGVPRPLAMKKESIQTRKRKPK-MPKTKSSSGSTVSGATSPTSLP---VSEN-ASTIKSEP  327
GATA-6_Human                  ECVNCGSIQTPLWRRDGTGHYLCNACGLYSKMNGLSRPLIKPQKR---VPSSRRLGLSCANCHTTTTTLWRRNAEGEPVCNACGLYMKLHGVPRPLAMKKEGIQTRKRKPKNINKSKTC--SGNSNNSIPMTPT---STSSN--SDDCSK  528
ENSBTAG00000005734_Cow        ECVNCGSIQTPLWRRDGTGHYLCNACGLYSKMNGLSRPLIKPQKR---VPSSRRLGLSCANCHTTTTTLWRRNAEGEPVCNACGLYMKLHGVPRPLAMKKEGIQTRKRKPKNINKSKAC--SGNSNNSVPMTPT---STSSN--ADDCSK  432
ENSMUSG00000005836_Mouse      ECVNCGSIQTPLWRRDGTGHYLCNACGLYSKMNGLSRPLIKPQKR---VPSSRRLGLSCANCHTTTTTLWRRNAEGEPVCNACGLYMKLHGVPRPLAMKKEGIQTRKRKPKNINKSKAC--SGNSSGSVPMTPT---SSSSN--SDDCTK  522
ENSRNOG00000023433_Rat        ECVNCGSIQTPLWRRDGTGHYLCNACGLYSKMNGLSRPLIKPQKR---VPSSRRLGLSCANCHTTTTTLWRRNAEGEPVCNACGLYMKLHGVFQPLSSQKESICWRKRVPDNIKKNKTD--SGNKSLPMCRTPT---RSAGN--TTSGDL  520
ENSTRUG00000012674_J_Puffer   ECVNCGSISTPLWRRDGTGHFLCNACGLYSKMNGLSRPLIKPQKR---TSTSRRIGLSCANCQTSTTTLWRRNAEGEPVCNACGLYTKLHGVPRPLAMKKEGIQTRKRKPKTLNKAKGSGISGNNS--VSMTPT---STSSS-NSEDCSK  448
ENSTNIG00000014837_T_Puffer   ECVNCGSISTPLWRRDGTGHFLCNACGLYSKMNGLSRPLIKPQKR---TSTSRRIGLSCANCQTSTTTLWRRNAEGEPVCNACGLYTKLHGVPRPLAMKKEGIQTRKRKPKTLNKTKGS--SGNNS--VSMTPT---STSSS-NSEDCSK  435
ENSDARG00000035831_Zebrafish  ECVNCGSMSTPLWRRDGTGHFLCNACGLYSKMNGLSRPLIKPQKR---MSSSRRIGLSCANCQTSTTTLWRRNAEGEPVCNACGLYTKLHGVPRPLAMKKEGIQTRKRKPKSLSKVKGS--SGISS--VPMTPT---SSSSS-NSEECTK  434
ENSXETG00000003144_Frog       ECVNCGSVQTPLWRRDGTGHYLCNACGLYSKMNGLSRPLIKPQKR---VPSSRRIGLACANCHTTTTTLWRRNTEGEPVCNACGLYMKLHGVPRPLAMKKEGIQTRKRKPKNLNKSKSSSSNGNSSHQIPMTPT---STTSSTNSDDCIK  458
FBgn0003117_Fly               ECVNCGAISTPLWRRDGTGHYLCNACGLYHKMNGMNRPLIKPSKRLVSATATRRMGLCCTNCGTRTTTLWRRNNDGEPVCNACGLYYKLHGVNRPLAMRKDGIQTRKRKPKKTGSGSAVGAGTGSGTGSTLEAIKECKEEHDLKPSLSLE  317
                              .......460.......470.......480.......490.......500.......510.......520.......530.......540.......550.......560.......570.......580.......590.......600
```

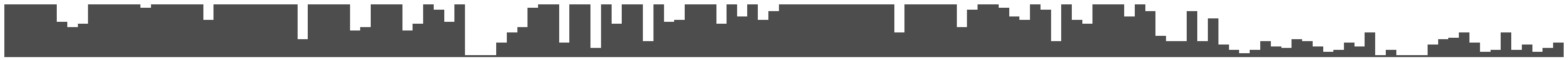

CLUSTAL 2.1 MULTIPLE SEQUENCE ALIGNMENT


```
                                                                                       .       :
GATA-4_Human                      EMRPIKTEPGLSSHYGHSSSVSQT-FSVSAMSGHGPSIHPVLSALKLSPQGYASPVSQSPQTSSKQDSWNSLVLADSHGDIITA------------------------------------------------------------------  442
ENSPTRG00000019993_Chimpanzee     EMRPIKTEPGLSSHYGHSSSVSQT-FSVSAMSGHGPSIHPVLSALKLSPQGYASPVSQSPQTSSKQDSWNSLVLADSHGDIITA------------------------------------------------------------------  399
ENSBTAG00000005425_Cow            EMRPIKTEPGLSSHYGPSSSLSQT-FSVSAMSGHGSSIHPVLSALKLSPQGYASSVSQSPQASSKQDPWNSLALADSHGDIITA------------------------------------------------------------------  442
ENSRNOG00000010708_Rat            EMRPIKTEPGLSSHYGHSSSMSQT-FST--VSGHGSSIHPVLSALKLSPQGYPSPVTQTSQASSKQDSWNSLVLADSHGDIITA------------------------------------------------------------------  441
ENSMUSG00000021944_Mouse          EMRPIKTEPGLSSHYGHSSSMSQT-FST--VSGHGPSIHPVLSALKLSPQGYASPVTQTSQASSKQDSWNSLVLADSHGDIITA------------------------------------------------------------------  442
ENSGALG00000016662_Chicken        EMRPIKTEPGLSSHYGHPSPISQA-FSVSAMSGHGSSIHPAISALKLSPQAYQSAISQSPQASSKQDSWNSLVLAENHGDIITA------------------------------------------------------------------  411
ENSXETG00000000673_Frog           EMRPIKIEPGLSPPYGHSSSLSQA-SVLSTITSHGSSSYP-MSSLKLSPQNHHSTINTSPQASSKHDSWNNLVLA---------------------------------------------------------------------------  394
ENSTRUG00000012112_J_Puffer       EPRHIKTEPDTHSLYTHHSAHAQ----ISALPAYMT-AHGGAIPLKMSPGGHT------GSSGSKAEPWNSLILA---------------------------------------------------------------------------  386
ENSTNIG00000016369_T_Puffer       EPRHIKTEPESHSLYSHHSTHAQSPLQISALPAYMA-THSGAIPLKMSPGSHG------GSSGSKAEPWNNLILA---------------------------------------------------------------------------  386
ENSDARG00000035759_Zebrafish      ETRPIKTEPDTTPLYTHHNIHTQ----VSAFPAYMG-SPS-------------------GSSSTKSEVWNSLILA---------------------------------------------------------------------------  352
GATA-5_Human                      SLASPVCPGPSMA--PQASGQEDDSLAPGHLEFKFEPEDFAFPSTAPSPQAGL-------RGALRQEAWCALALA---------------------------------------------------------------------------  397
ENSPTRG00000013711_Chimpanzee     SLASPVCPGPSMA--PQASGQEDDSLAPGHLEFKFEPEDFAFPSTAPSPQAGL-------RGALRQEAWCALALA---------------------------------------------------------------------------  397
ENSCAFG00000012709_Dog            SLASPSCPGSSIT--SQAPGPVDDPLAPSHLEFKFEPEDFAFPSAALGPQAGL-------GGTLRQEAWCALALA---------------------------------------------------------------------------  400
ENSMUSG00000015627_Mouse          SLASPVCAGPTIT--SQASSPADESLASSHLEFKFEPEDFAFTSSSMSPQAGL-------SGVLRQETWCALALA---------------------------------------------------------------------------  404
ENSRNOG00000036732_Rat            SLASPGCAGPTIT--SQASSPADESLASSHLEFKFEPEDFVFTSSSLSPQAGL-------SGVLRQEAWCALALA---------------------------------------------------------------------------  404
ENSGALG00000005352_Chicken        STTS-QYPGQGIVSVSQAQSQSDEALAGG--EFKFEPEDYPFSPSSMAPQPGL-------SVPLRQDSWCALALA---------------------------------------------------------------------------  391
ENSTNIG00000002769_T_Puffer       SSAPSPYAGQTLAAATQTASHLD-RVG--QVDIKY--EDYPFAPASISSP----------------HSWCALSQA---------------------------------------------------------------------------  392
ENSDARG00000017821_Zebrafish      SIAASPYAGQTVVSVTQASTQLD-SASSAHVDIKY--EDYTYTPTSIAPQ----------------NSWCALSQA---------------------------------------------------------------------------  383
GATA-6_Human                      NTSPTTQPTASGAGAPVMTGAGE-STNPENSELKYSGQDGLYIGVSLASPAEV-------TSSVRPDSWCALALA---------------------------------------------------------------------------  595
ENSBTAG00000005734_Cow            NTSPTTQPAASGAGGSVMSGAGD-SANPENSELKYSGQDGLYIGVSLASPAEV-------TSSVRQDSWCALALA---------------------------------------------------------------------------  499
ENSMUSG00000005836_Mouse          NTSPSTQATTSGVGASVMSAVGE-NANPENSDLKYSGQDGLYIGVSLSSPAEV-------TSSVRQDSWCALALA---------------------------------------------------------------------------  589
ENSRNOG00000023433_Rat            NQSPPPQSTPYEVGASVMSAVGE-SANPENSDLKYSGQDGLYIGVSLSSPAEV-------TSSVRQDSWCALALA---------------------------------------------------------------------------  587
ENSTRUG00000012674_J_Puffer       TSSPPGQG--SGVSSSVLSSSGE-GTGSS-SAVKYPGQDGLYTSVGLSQASDV-------AS-VRGEPWCPMALA---------------------------------------------------------------------------  511
ENSTNIG00000014837_T_Puffer       -SSPSGQG--SGVSSSVLSSSGE-GTGSS-SAVKYPGQDGLYSSVGLSQAADV-------AS-VRGEPWCPMALA---------------------------------------------------------------------------  497
ENSDARG00000035831_Zebrafish      NSPSSTQVPVTSVNSSTLVGQVD-VPGTTGSMVKFPGQESLYTNVGLTSSADV-------ASSVRGDSWCPMALA---------------------------------------------------------------------------  501
ENSXETG00000003144_Frog           NGSPSQNT-APVVASSLMSTQTD-STSPNSNTLKYTGQDGLYSAVSLSSASEV-------AASVRQDSWCALALA---------------------------------------------------------------------------  524
FBgn0003117_Fly                   RHSLSKLHTDMKSGTSSSSTLMGHHSAQQQQQQQQQQQQQQQQQQQQSAHQQCFPLYGQTTTQQQHQQHGHSMTSSSGQAHLSARHLHGAAGTQLYTPGSSSGGGSASAYTSHSAETPALSNGTPSPHYQHHHHLGGTHGHHVTAAAAHH  467
                                  .......610.......620.......630.......640.......650.......660.......670.......680.......690.......700.......710.......720.......730.......740.......750
```

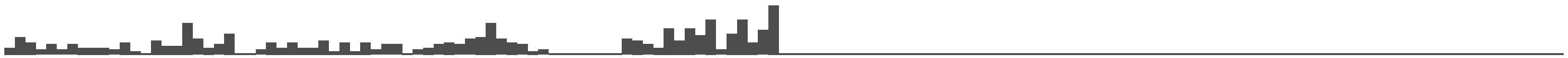

```
GATA-4_Human                      -------------------------------------------------------------------------  442
ENSPTRG00000019993_Chimpanzee     -------------------------------------------------------------------------  399
ENSBTAG00000005425_Cow            -------------------------------------------------------------------------  442
ENSRNOG00000010708_Rat            -------------------------------------------------------------------------  441
ENSMUSG00000021944_Mouse          -------------------------------------------------------------------------  442
ENSGALG00000016662_Chicken        -------------------------------------------------------------------------  411
ENSXETG00000000673_Frog           -------------------------------------------------------------------------  394
ENSTRUG00000012112_J_Puffer       -------------------------------------------------------------------------  386
ENSTNIG00000016369_T_Puffer       -------------------------------------------------------------------------  386
ENSDARG00000035759_Zebrafish      -------------------------------------------------------------------------  352
GATA-5_Human                      -------------------------------------------------------------------------  397
ENSPTRG00000013711_Chimpanzee     -------------------------------------------------------------------------  397
ENSCAFG00000012709_Dog            -------------------------------------------------------------------------  400
ENSMUSG00000015627_Mouse          -------------------------------------------------------------------------  404
ENSRNOG00000036732_Rat            -------------------------------------------------------------------------  404
ENSGALG00000005352_Chicken        -------------------------------------------------------------------------  391
ENSTNIG00000002769_T_Puffer       -------------------------------------------------------------------------  392
ENSDARG00000017821_Zebrafish      -------------------------------------------------------------------------  383
GATA-6_Human                      -------------------------------------------------------------------------  595
ENSBTAG00000005734_Cow            -------------------------------------------------------------------------  499
ENSMUSG00000005836_Mouse          -------------------------------------------------------------------------  589
ENSRNOG00000023433_Rat            -------------------------------------------------------------------------  587
ENSTRUG00000012674_J_Puffer       -------------------------------------------------------------------------  511
ENSTNIG00000014837_T_Puffer       -------------------------------------------------------------------------  497
ENSDARG00000035831_Zebrafish      -------------------------------------------------------------------------  501
ENSXETG00000003144_Frog           -------------------------------------------------------------------------  524
FBgn0003117_Fly                   HFHAAAAVAAYGVKTEASATNYDYVNNCYFGGTFGALGGAATTTAMAGGAASELAGYHHQHNVIQAAKLMATS  540
                                  .......760.......770.......780.......790.......800.......810.......820...
```
