# Supplementary material for: Identification of Functional Mutations in GATA4 in Patients with Congenital Heart Disease
Source: PLoS One. 2013 Apr 23;8(4):e62138. doi: 10.1371/journal.pone.0062138 (PMC3633926; doi:10.1371/journal.pone.0062138)
Supplement: Table S1 — GATA4 primers used in PCR amplification. (DOCX) [file pone.0062138.s003.docx]

**Table S1 *GATA4* primers used in PCR amplification**

| Exon | Forward (5’ to 3’) | Reverse (5’ to 3’) | Product length |
| --- | --- | --- | --- |
| Exon1  Exon2  Exon3  Exon4  Exon5  Exon6 | CTGGGCCTGTCCTACCTCCA  tccaaggaAAGGGCATTGTT  TCTCATGCAGGGTCGTTagg  TTCTCGcagcaggtgtgtgt  ccATTAGCTTGCACCCATCC  ATCACCGGGAtcaggagaaa | GGTCCCCGGGAAGGAGAAG  CGATGCACACCCtcaagttc  GTTGAAagccccttccaaat  ATGTCCGATGCTGTCACCAC  TTCCTAGCGCAGAGGGTAGC  GGAGTCATTaccaggcagagg | 779  385  393  298  382  451 |
